# Assessing dental professionals’ understanding of tobacco prevention and control: a qualitative study in Västerbotten County, Sweden

**DOI:** 10.1038/bdjopen.2016.9

**Published:** 2016-12-23

**Authors:** Raman Preet, Nausheen Khan, Yulia Blomstedt, Maria Nilsson, Jennifer Stewart Williams

**Affiliations:** 1 Unit of Epidemiology and Global Health, Department of Public Health and Clinical Medicine, Faculty of Medicine, Umeå University, Umeå, Sweden; 2 Laboratory of Muscle Biology, Anatomy, Integrative Medical Biology, Umeå University, Umeå, Sweden; 3 Priority Research Centre for Generational Health and Ageing, Faculty of Health and Medicine, University of Newcastle, Newcastle, NSW, Australia

## Abstract

**Aim:**

To assess dental professionals’ understanding of tobacco prevention and control.

**Materials and methods:**

In Sweden dental hygienists receive training in tobacco prevention and control. The study setting is Västerbotton County in the north of Sweden where a number of successful tobacco control initiatives have been established. A purposeful sample comprising five male and four female dental professionals and trainees was selected. Data were collected through in-depth semi-structured individual interviews and analysed using content analysis.

**Results:**

Informants acknowledged limited adherence to tobacco prevention. They were not confident of their knowledge of tobacco and non-communicable disease prevention and had limited awareness of global oral health policies. Reasons for poor adherence included professional fragmentation, lack of training, and the absence of reimbursement for time spent on prevention activities.

**Discussion:**

The success of efforts to reduce smoking in Västerbotton County is attributed to the network of local public health initiatives with very limited involvement by local dental professionals.

**Conclusions:**

The findings highlight the need to more actively engage the dental workforce in tobacco control and prevention. Moreover, it is important to recognise that dental professionals can be public health advocates for tobacco control and prevention at global, national and local levels.

## Introduction

The tobacco epidemic is a global epidemic that each year kills approximately six million people and has substantial social and economic costs. It is expected that tobacco will cause a billion deaths this century if the World Health Organization’s Framework Convention on Tobacco Control (WHO FCTC) is not fully implemented.^1^ In Sweden, smoking prevalence has declined in recent years and is among the lowest in the industrialised world.^2^ Yet it is estimated that almost 54,500 lives will be lost in Sweden because of tobacco use by the year 2040.^3^ Oral health is part of general health and is positioned within the global health policy agenda on tobacco control.^4–6^ The role of dental professionals in combating tobacco smoking is supported by Fédération Dentaire Internationale (FDI) World Dental Association and the World Health Organization (WHO).^7^ Smoking cessation is one of the most important health services that dental personnel can provide.^4–6^ Dentists are well-placed as agents for tobacco cessation and general health promotion;^4^ however the adoption of these activities in practice, has been slow.^8^

The dental workforce in Sweden is unique in comprising a mix of professionals—dentists (multi-specialties), dental hygienists, dental nurses and dental technicians.^9^ Dental hygienists are the only dental professionals with specific training in tobacco prevention and control as part of their core oral health education.^9^ The dentist, hygienist and nurse have direct patient contact. About 80% of the adult population in Sweden receive dental services every two years, yet the potential for tobacco prevention activities by dental professionals is not fully realised.^10^ It is often assumed that the dental profession in Sweden is sufficiently well informed and trained to engage in tobacco prevention and control, but there is little evidence of this. We ask the question: can Sweden’s success in tobacco control and prevention be attributed to the efforts of dental professionals? The purpose of this qualitative study is to help answer this question from a local perspective.

In Sweden, public health education for disease prevention and health promotion is delivered locally. Here we investigate health promotion activities from the perspective of dental professionals in Umeå, Västerbotten County where one of the country’s four dental schools is located and a number of successful tobacco prevention and control programs have been implemented. The aim of our qualitative study was to assess dental professionals’ understanding of tobacco prevention and control in this local setting.

## Materials and methods

### Study setting

Umeå is the biggest city in northern Sweden and is well-known for its successes in tobacco control and other health intervention programmes. Two notable examples are the ‘*Tobacco-Free Duo’*^11^ and the ‘*Smoking cessation*’ programme.^12^ Tobacco-Free Duo is a long term intervention program with adolescent-adult partnership on a community level aimed at reducing tobacco use amongst youth.^11^ Both programmes utilise the services of dental hygienists within teams of multidisciplinary professionals.

Another internationally acclaimed population-based programme, the Västerbotten Intervention Programme^13^ was initiated in 1985 to address rising mortality from cardiovascular disease in the County, which, at the time, had one of the highest rates of cardiovascular disease in Sweden. The Västerbotten Intervention Programme aims to reach all adults, at ages 40, 50 and 60 years, through participation in systematic risk factor screening in relation to cardiovascular disease and diabetes. Participants receive health education regarding tobacco use and individual counselling for healthy lifestyle behaviours.^13^ A recent community health intervention, ‘*Salut’*, has a multi-sectoral family centered approach to child health promotion and illness prevention. Salut provides epidemiological surveillance and health promotion interventions for all expectant parents and their children, up to 18 years of age, in Västerbotten. Salut has a dental component as an integral part.^14^ Building on the success of these programmes, the Västerbotten County Council’s vision is to have the best health and the healthiest population in the world by 2020.^15^

### Study design

In-depth semi-structured interviews were conducted individually with dental personnel to capture their knowledge, views, perceptions and experiences regarding tobacco use, control and prevention, at both local and global levels. Before commencing this study, the first author undertook a short project which assessed dental students’ awareness of tobacco control and prevention, as part of a Masters of Public Health degree awarded in 2009.^16^ For that earlier project, six final semester, soon to graduate, dental students were interviewed using a thematic guide which was based on the theory of globalisation^17^ that defines globalisation as movement of people, information and commodities in global and transnational spaces. The first and second authors revised this guide in line with the literature to allow for the different mix of dental personnel in this study. The guide was then piloted-tested and modified. All interviews were conducted using the following four themes within the guide: (i) individual, (ii) professional, (iii) regional/national and (iv) global. See Appendix 1.

### Study population

A purposeful sample of dental personnel was selected in order to cover a range of attributes in relation to age, sex, professional category and practice settings^18^. The final sample size was determined when data saturation was reached at the ninth interview. The nine informants in the sample comprised seven dental personnel and two dental students from Umeå University. All those approached and asked to participate agreed to be interviewed. Table 1 gives the breakdown of informants by professional category, sex and age.

### Data collection

Information about the study was distributed by email. Informants gave written consent to participate. During follow-up phone calls appointments were made for the interviews which were conducted in venues of choice, which were comfortable and familiar for all. Participation was voluntary and no financial incentives were provided. Primary data were collected through semi-structured in-depth interviews, probing with open-ended questions consistent with the thematic guide.

The second author conducted all the interviews which were between 30 and 60 min in duration. Informants were reassured of confidentiality and anonymity. The interviews were transcribed verbatim within one or two days of being conducted. By the time data collection ceased at the ninth interview, there was ample coverage of different viewpoints. Although most informants were fluent in English, this was their second language. The services of a translator were utilised for one informant who could communicate only in Swedish.

### Data analysis

The process of analysis was guided by the thematic guide. Data were analysed using content analysis where the narrative text obtained from all interviews was deciphered to derive meaningful units.^19^ The first and second author coded in parallel and organised the information into emerging patterns which formed the basis of several categories. The results were grouped in themes, as discussed below. All authors were in agreement and triangulation was used to cross-verify and enhance the validity of the findings.^18^

## Results

**Figure 1:**
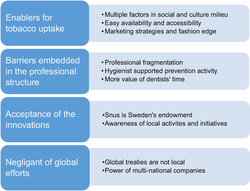
Themes and sub-themes for understanding of tobacco prevention and control.

Informants shared many perceptions and opinions. Four themes emerged from the data: (a) enablers for tobacco uptake, (b) limitations embedded in the professional structure, (c) acceptance of national innovations and (d) unawareness of global efforts. The findings are illustrated by direct quotes, in italics. Results are under sub-headings within themes, as shown in Figure 1.

### Enablers for tobacco uptake

#### Multiple factors in the social and culture milieu

The act of tobacco consumption by an individual cannot be attributed to his or her personal choice alone. All the informants talked about this in their interviews. Giving in to peer pressure dynamics to gain acceptance in the group, as well as multiple factors that operate in the larger social cultural milieu were seen as being strong influences on decisions to use tobacco.

“...*Back then in 1950s…. probably yeah I think it was an individual choice and peer pressure. Nowadays the individual knows that it is bad. It is not an individual choice. I think it should be more social”.*

*“They will use it irrespective of anything because if it’s an illegal substance you will try to use it and if it is socially and culturally acceptable, then also the people will use it”.*

#### Easy availability and accessibility

Easy availability and accessibility of tobacco in the market paves way for addiction. All informants were of the opinion that youth smoking is on the rise globally. The placement of tobacco at very visible and easy to locate places in shops in Sweden was seen as a ‘push factor’ encouraging the uptake of smoking, tobacco and snus.

*“It is very easy to get it, I think that makes it very accessible and affects people”*

#### Marketing strategies and fashion edge

Media-based marketing strategies by tobacco companies were seen as being responsible for imparting a ‘fashion edge’ on tobacco, despite the growing public awareness of its ill effects on health. The warning messages on tobacco packages were discussed with mixed feelings. Informants said that over time the messages about tobacco have become more alarming with specific information being given about the health dangers of tobacco. However, it was also thought that the public has been somewhat desensitised to these warnings, and that they no longer have the same impact as they did when they were first introduced.

*“In Europe in general, people have heard it so much that they are just tired of hearing that tobacco is bad for you. I think it was a few years ago that it was an active debate and on television but nowadays there are no commercials about anti smoking anymore”*

### Limitations embedded in the professional structure

Informants talked about different issues that limit their involvement in tobacco prevention activities. Three different categories were identified as professional fragmentation, hygienist supported prevention activity, and the value of the dentists’ time.

#### Professional fragmentation

The dental workforce in Sweden is unique in the way in which it is structured with defined duties for each sub-specialty. As an activity, counselling and health education was understood to be the responsibility of one group only, the hygienists. Moreover, there was broad agreement that dentists’ skills were intended for treatment procedures rather than for prevention and education.

*“The dental hygienist’s job is more towards telling about taking care of teeth and motivating patients, whereas dentists talk less and do more”*

#### Hygienist supported prevention activity

The provision of preventive messages and counselling on tobacco use was seen as important. Informants expressed the view that tobacco control and prevention activities were the exclusive responsibility of dental hygienists. With the exception of the hygienists, informants discussed the lack of training that they had in this regard during their professional dental education. The students in the study said that they were not confident that they fully understood the general health risks of tobacco use and they were therefore not confident about providing this information to their patients. However, they expressed curiosity and interest in knowing more.

*“We have not been taught about how to tell or inform patients on how to quit. Dentists can play a role but need more knowledge”*

#### Value of the dentists’ time

All informants affirmed that the dentist’s time in treating and providing care is highly valuable. The lack of financial compensation for time spent on prevention activities was a major disincentive for dentists to provide education in usual practice. In Sweden the dentist and hygienist work together in the clinical setting, so some saw that there was little need for dentists to take responsibility for these activities.

“*Both (dentist and hygienist) have an important role. It’s important that dentists inform the patient. More confidence in the dentists. Same time very important that hygienist tries a behavior change because a dentist time is valuable, economically valuable”.*

### Acceptance of national innovations

#### Snus is Sweden’s endowment

Informants reflected vividly on the use of ‘snus’ which is very common in Sweden. They expressed the view that snus is an acceptable alternative to tobacco smoking as they understood that it is less harmful than tobacco smoking. However, concerns were raised in relation to limited available evidence on the effects, harmful and otherwise, of consuming snus. Informants felt that more population-based research is needed. On the short and long term effects of snus.

*Snus is very widespread. There are some mucosal changes. We need more population studies*.”

*“Snus-not good. If we compare everybody knows cigarette is more dangerous. Snus-less harmful”*.

#### Awareness of local activities and successes

The fact that Västerbotten County is a leader in tobacco prevention nationally was well-known and celebrated amongst the informants. It was commonly believed that programmes like ‘*Tobacco Free Duo’* had worked well and were effective. Some of the informants and their families were recipients who had benefited from these or other similar health promotion programmes.

*“Yeah. They have this about two generations for example- a child and mother. They can write an agreement. I did it with my son and it worked. He doesn’t smoke. He is 28 years old now.”*

### Unawareness of global efforts

#### Global is not local

During the interviews informants were asked about the WHO FCTC international treaty. The purpose was to engage their views on global issues. Even though Sweden is a signatory, all informants were unaware of this treaty. They were fully aware of Sweden’s success in tobacco control but were unsure of its global implications. A common view expressed by informants was that the global treaties were of no help in the Swedish context.

*“Don’t know about FCTC. But, it seems good to what it is doing as told now but not sure what it means for Sweden”*

#### Power of multinational companies

Tobacco addiction was discussed as a global health problem but there was a general indifference about tobacco cultivation and its harmful impacts, with the exception of one informant who linked it to pesticides, deforestation and low-income countries. Informants said that tobacco control related issues require attention at all levels—individual, societal, professional, political, national and international. The view was expressed that a multifaceted approach would be one way of tackling the powerful business operations of the multinational tobacco companies.

“*Government can work on companies. Multinational companies are pumping money in the development of developing countries and they are powerful”.*

*“Nine million people in Sweden can’t change the world. But it can set an example”*.

## Discussion

Research shows that compliance with guidelines recommending that oral health providers play a role in tobacco cessation and prevention is poor, especially among dentists.^7^ In Sweden, dentistry has a broad skill base divided into sub-specialties, with distinct roles and responsibilities that are intended to map with skills and training. This professional delineation offers potential for delivering targeted oral health promotion such as tobacco control and prevention. However, in this qualitative study, informants acknowledged limited adherence to tobacco prevention and control activities. This was an unexpected finding given that the dental workforce in Sweden is well-placed to undertake health promotion, and also because the study was set in an area in which there have been major advances in tobacco control and prevention through successful health promotion programmes.

Fragmentation between dental sub-specialties may not be conducive to oral health promotion. Although Sweden is well-known for its egalitarian culture, there was an implied ordering of roles and responsibilities. Dentists took the view that their more highly valued role was one of treatment and that tobacco prevention and education are the hygienist’s responsibility. This is somewhat ironical given that dentists in this study and in other contexts^5,6^ saw tobacco control and prevention as being a very important service that should be offered by the profession. The translation to practice was not evident.^8^

Although they recognised that tobacco control and prevention was expected of them, hygienists were not confident of their ability to undertake these activities. However fragmentation and disintegration of professional responsibilities among dentists and dental hygienists is characteristic of the Swedish dental workforce and may not occur in other countries. In India, for example, dentists are trained to perform different roles including that of the hygienist^20^ and general health promotion.

Dentists’ treatment skills were valued more highly than education skills. However a Cochrane review found that advice from medically trained health professionals has a greater impact than advice from non-medical health professionals. Short clear advice from a medical doctor about quitting smoking increases the probability that a smoker will remain a non-smoker for at least 12 months.^21^ It was suggested that the lack of monetary compensation for preventive services was a disincentive for dentists to integrate prevention and treatment activities into everyday practice.^4,5^ It may well be worthwhile to investigate policy options that offer dentists monetary compensation for prevention activities, particularly in settings where oral health education is not otherwise available or accessible.^20^

Achievements in tobacco control and prevention in northern Sweden appear to be related to local public health initiatives. All informants were aware of and praised local and national tobacco control and prevention activities. Some had personally benefitted from these programmes and, although they were not identified in this study, other dental professionals in the area participated in community smoking cessation activities conducted in the County.^22^ However the translation of good practice in Sweden to other settings is not assured. Every workforce context is different.^20^

The subject of snus was introduced in the interviews. Snus is a moist powder smokeless tobacco product placed commonly under the upper lip and is well-known as a Swedish nicotine product. Other types of smokeless tobacco, such as chewing tobacco, are used to a limited extent in Sweden. Snus has been used in Sweden for more than 200 years, with a higher prevalence in northern Sweden compared with the rest of the country. The informants in this study did not consider snus as harmful as smoking, although they also expressed skepticism about the long term risks. Harmful effects from snus are often compared with those from smoking. The comparison easily becomes skewed as there are few things as dangerous to health as smoking. The health risks from snus should rather be compared with not using any form of tobacco. However, there is a great need for an increased research focus on snus and its health implications, as this has been limited in the past. Snus has a strong presence, especially in northern Sweden, and a greater scientific basis of health effects from snus is an important part of the work to change people's view on snus. Snus cessation is usually carried out with the same methods used in smoking cessation, and would also benefit from a larger evidence base.

Global awareness on tobacco control and knowledge on WHO FCTC was a key aspect of the study. With FCTC, WHO is helping nations in tobacco control as needed. But, global is not local. Informants were not aware of global treaties or efforts on tobacco control. Raising awareness about the global policy among dental professionals is important but policies need to be translated into practice. For example, the objectives of 2013 ‘*The Stockholm Declaration for Global Health’*^23^ are very relevant for oral health; in particular in building capacity for leadership through education and training at the grass roots level. But the way forward will be through interdisciplinary inter-professional engagement at all levels.^24^

Informants displayed a lack of confidence with regard to undertaking broader health promotion and education activities. This was disappointing given the well-recognised importance of tackling the causes of non-communicable diseases (NCDs) and oral diseases from a common base.^25–27^ The WHO declares, ‘Oral health promotion and oral disease prevention should embrace what is termed ‘the common risk factor approach (CRFA),’^28^ leading to the integration of oral health promotion into broader health promotion’.^29^ Social and behavioural risk factors and social determinants are common in both general and oral health.^27–29^ It is important to ensure that in order to tackle the individual act of consuming tobacco, there is a need to address socio-economic and cultural determinants under an integrated CRFA.^30^

### Strengths and limitations

The study results are derived from a small sample of dental professionals and the results are not representative of all dental health professionals in Umeå or Västerbotten County. If, for example, informants had included dental professionals who had been members of local multidisciplinary health promotion teams, then responses may have been different. A strength of the study is that although the results are context specific, they provide a platform for understanding these important public health issues and the sorts of questions that need to be addressed in other settings, both in Sweden and internationally.

We are not aware of any selection bias. The sample included informants with a range of attributes and professional backgrounds. We did not have prior knowledge regarding the way in which informants were likely to respond to the interview questions. Although the sample size was small, data saturation was reached.

Our study sample was not intended to provide results which could be generalised to a broader population of dental professionals. The aim was to provide sufficient depth to better assess dental professionals’ understanding of tobacco prevention and control in this setting in Västerbotten County, Sweden. The data support our conclusions and the conclusions do not extend beyond what the data can support.

### Policy implications

The results of this qualitative study suggest the need for further investigation, initially through research and policy formulation, and later through implementation and evaluation. Structures are in place in the dental profession in Sweden but the workforce culture and attitudes need honest assessment and evaluation. Dental professionals must practice tobacco control activities and policy makers need to adjust remuneration accordingly. It is important to recognise and value integrated oral and health promotion activities at the individual and population level. There are benefits to be gained and shared by providers and recipients but this requires commitment and collaboration by health professionals, individuals and communities.

## Conclusions

It is important to ensure that dental professionals are given opportunities to become advocates for their profession and signal the role that they can play in tobacco prevention and control as well as in NCD health promotion at global, national and local levels. This must be done in collaboration with other health professionals. Sweden is well-placed to be a leader in addressing this global health priority.

## Figures and Tables

**Table 1:** Characteristics of study informants

*Informant No.*	*Professional category*	*Sex*	*Age (years)*	*Total number*
1, 2	Dental students	Male	24, 26	2
3, 4	Teacher/Specialist	Male	53, 32	2
5	Hygienist	Female	30	1
6	Nurse	Female	55	1
7	Dental Assistant	Female	54	1
8	Private dentist	Male	31	1
9	Dental Secretary	Female	42	1
Total				9

## Appendix 1

1. **Individual**—If tobacco use an individual choice or is influenced by other factors?
  - Personal experience about tobacco and its uses. Have you ever used it? If yes, what was it that made you use? If No, How did you resist it?
  - What do you think availability of tobacco? Is easy access is a problem?
  - Any social and cultural factors you think, play a role.
  - Any lectures given in tobacco control like—cessation skills etc.
  - Who do you know advises on smoking cessation, a dentist or hygienist?
  - Share your opinion if dentist can play a role in local level activities.
  - What is the awareness about tobacco control in Västerbotten and in Sweden?
  - What is your opinion about Swedish Snus?
  - Does media play a role in tobacco activities? (Endorsing or reducing)
  - Tobacco production is also injurious to health because of the way it’s cultivated.
  - Do you know anything about this aspect?
  - Do you think tobacco issues need to be dealt at all levels like political, societal, professional, and governmental? Any reasons you can share.
  - Do you know about FCTC? If does not know, tell the respondent and ask if this kind of treaty can play any role in bringing all countries together and deal with this man-made epidemic
2. **Professional**—Role of dentists in this preventable cause of disease.
  - Any lectures given in tobacco control like—cessation skills etc.
  - Who do you know advises on smoking cessation, a dentist or hygienist?
  - Share your opinion if dentist can play a role in local level activities.
  - What is the awareness about tobacco control in Västerbotten and in Sweden?
  - What is your opinion about Swedish Snus?
  - Does media play a role in tobacco activities? (Endorsing or reducing)
  - Tobacco production is also injurious to health because of the way it’s cultivated.
  - Do you know anything about this aspect?
  - Do you think tobacco issues need to be dealt at all levels like political, societal, professional, and governmental? Any reasons you can share.
  - Do you know about FCTC? If does not know, tell the respondent and ask if this kind of treaty can play any role in bringing all countries together and deal with this man-made epidemic
3. **Regional/ National**—Tobacco control in Sweden
  - What is the awareness about tobacco control in Västerbotten and in Sweden?
  - What is your opinion about Swedish Snus?
  - Does media play a role in tobacco activities? (Endorsing or reducing)
  - Tobacco production is also injurious to health because of the way it’s cultivated.
  - Do you know anything about this aspect?
  - Do you think tobacco issues need to be dealt at all levels like political, societal, professional, and governmental? Any reasons you can share.
  - Do you know about FCTC? If does not know, tell the respondent and ask if this kind of treaty can play any role in bringing all countries together and deal with this man-made epidemic
4. **Global**—Tobacco Control Activities
  - Tobacco production is also injurious to health because of the way it’s cultivated.
  - Do you know anything about this aspect?
  - Do you think tobacco issues need to be dealt at all levels like political, societal, professional, and governmental? Any reasons you can share.
  - Do you know about FCTC? If does not know, tell the respondent and ask if this kind of treaty can play any role in bringing all countries together and deal with this man-made epidemic
